# Intended and Unintended Consequences of a Community-Based Fresh Fruit and Vegetable Dietary Intervention on the Flathead Reservation of the Confederated Salish and Kootenai Tribes

**DOI:** 10.3389/fpubh.2020.00331

**Published:** 2020-08-07

**Authors:** Selena Ahmed, Virgil Dupuis, Michael Tyron, MaryAnn Running Crane, Teresa Garvin, Michael Pierre, Carmen Byker Shanks

**Affiliations:** ^1^Food and Health Lab, Sustainable Food Systems Program, Department of Health and Human Development, Montana State University, Bozeman, MT, United States; ^2^Extension, Salish Kootenai College, Pablo, MT, United States; ^3^Gretchen Swanson Center for Nutrition, Omaha, NE, United States; ^4^Flathead Food Distribution Program on Indian Reservations, Pablo, MT, United States

**Keywords:** dietary intervention, food and nutrition education, community-engaged research, dietary quality, co-design

## Abstract

Tribal communities in the United States face disparities to accessing healthy foods including high-quality produce. A six-week fresh fruit and vegetable (FV) dietary intervention, *Eat Fresh*, was co-designed with a Community Advisory Board of local food and nutrition stakeholders on the Flathead Reservation of the Confederated Salish and Kootenai Tribes in Montana. *Eat Fresh* was implemented as a pilot study with low-income participants (*n* = 19) enrolled in the Food Distribution Program on Indian Reservations toward improving dietary quality and perceptions of well-being. We evaluated *Eat Fresh* at pre- and post-intervention on the basis on food procurement practices, dietary quality using the Healthy Eating Index (HEI), Body Mass Index (BMI), blood pressure, and participant perceptions of health. Participants reported consuming a greater number of types of FVs daily during the intervention (*p* < 0.005 for fruits and *p* > 0.19 for vegetables). Overall, participants found *Eat Fresh* moderately challenging to adhere to with the main barriers being access to ingredients in recipes (39.51% of responses), time constraints to cook (35.80%), and lack of financial resources (33.33%). Dietary quality improved during the intervention from a mean HEI score of 48.82 (± 11.88) out of 100–56.92 (± 11.88; (*p* > 0.12). HEI scores for fruit consumption significantly increased (*p* < 0.05) from 1.69 (out of 5 points) during the pre-intervention to 2.96 during the post-intervention. BMI and blood pressure increased for several participants, highlighting an unintended consequence. Most participants responded that FV consumption made them feel either very good (51.16%) or good about their health (43.02%) with the majority (83%) perceiving an improvement in energy. Findings of this pilot study highlight both intended and unintended consequences of a dietary intervention that provide lessons in co-designing community-based programs.

## Introduction

Diet-related chronic health challenges are a primary risk factor of disease globally (1) while also being a leading preventable cause of death (2). Numerous studies have shown that a notable percentage of diet-related chronic challenges including cardiovascular disease, hypertension, cancer, Type II diabetes, and weight gain are preventable over time with healthy lifestyles that are supported by food environments that provide access to healthy and affordable foods (3–8). The food environment is the consumer interface of the food system that influences the availability, affordability, convenience, and desirability of foods and, subsequent food choices as well as health outcomes (4, 9). A growing body of evidence supports that residents of tribal and rural communities face disparities in their food environments to accessing healthy, affordable, convenient, and desirable foods (10–16).

Despite global and national efforts to improve dietary quality, diet-related chronic diseases, and food insecurity represent critical health disparities in the United States among Native American, African American, Hispanic, and Asian populations (17–22). For example, Type II diabetes has been shown to be epidemic among Native American populations with a higher rate of prevalence than any other ethnic group in the United States (19). In addition, Native American adults are twice as likely to be food insecure compared to non-Hispanic whites (22).

The diet-related health disparities observed in Native American communities are linked to food environment disparities that are influenced by multiple socio-ecological factors (23–25). Current food environments in tribal and rural communities in the United States are characterized by the prevalence of relatively affordable ultra-processed foods high in refined sugars, saturated fats, and salt (18) while having barriers to accessing high-quality produce (11–14, 16). Food environments where processed foods are more accessible, affordable, and desirable than fresh fruits and vegetables are associated with dietary patterns of overall reduced consumption of healthy foods (26) coupled with increased consumption of unhealthy high in saturated fats, sugar, and salt (18). Alternatively, the daily consumption of fruits and vegetables is integral for human nutrition and is associated with reduced risk of obesity and diet-related chronic disease (27). Increasingly, plant-based diets are recognized for their contribution to supporting both human and planetary health (28). However, accessing fresh, desirable, and high-quality fruits, and vegetables is a food environment disparity in tribal communities in the United States including on the Flathead Reservation of the Confederated Salish and Kootenai Tribes (hereafter “the Flathead Reservation”) in the rural state of Montana in the United States (11–16).

In order to address the aforementioned food environment and diet-related disparities in tribal communities, our research team co-designed a community-based fresh fruit and vegetable (FV) dietary intervention, titled *Eat Fresh*, with a Community Advisory Board of local food and nutrition stakeholders. We implemented *Eat Fresh* as a pilot study with low-income households on the Flathead Reservation of the Confederated Salish and Kootenai Tribes (hereafter Flathead Reservation). We used the Delphi method with the Community Advisory Board of local food and nutrition stakeholders on the Flathead Reservation to determine priorities of a dietary intervention research on prevention of obesity and diet-related disease as well as its cultural appropriateness and feasibility. *Eat Fresh* was co-designed to enhance consumption of fresh fruits and vegetables in order to improve dietary quality of participants by removing food environment barriers to accessing fresh fruits and vegetables and providing food, nutrition, and cooking education on produce to enhance its consumption. The overall research question of this study is: *What are the effects of a fruit and vegetable (FV) dietary intervention on dietary and health measures (including food procurement practices, dietary quality, Body Mass Index (BMI), blood pressure, and participant perceptions of health)?* Our overall goal is to apply findings to co-design future interventions and evidence-based public health community programs that enhance dietary quality and food sovereignty on the Flathead Reservation by improving access to fresh plant-based foods that are affordable, convenient, desirable, and sustainable.

## Methods

### Study Site

The *Eat Fresh* dietary intervention was co-designed and carried out as a pilot study on the Flathead Reservation of the Confederated Salish and Kootenai Tribes (hereafter Flathead Reservation) located in Northwest Montana in the United States. The Flathead Reservation is home to Bitterroot Salish, Upper Pend d'Oreille, and the Kootenai tribes and comprises of ~1.3 million acres of which ~768,000 acres belong to the Tribes and individual households with tribal affiliation (29). There are eight recognized townships on the Flathead Reservation including Arlee, Mission, Hot Springs, Ronan, Pablo, Polson, Elmo, and Charlo. The Flathead Reservation is home to ~28,993 residents of which 7,791 residents has a tribal designation (29).

### Co-design of the *Eat Fresh* Intervention

The intervention was co-designed in collaboration with a Community Advisory Board of local food and nutrition stakeholders on the Flathead Reservation coupled with the study team's previous experiences and research findings in the community. The study team was comprised of researchers and student trainees from Salish Kootenai College (a tribal college) and Montana State University. The study team and the Community Advisory Board includes tribal and non-tribal members.

The Community Advisory Board comprised of 15 food and nutrition stakeholders that live and work on the Flathead Reservation including Tribal elders, educators, enterprise representatives, clinical practitioners, and policy-makers, including a member of the Tribal Council. Selection of members of the Community Advisory Board was decided by the research team on the basis of previous experiences in the community as well as a snowballing approach of consultation with experts in the community in the areas of food and nutrition. In addition, a local community research coordinator with tribal affiliation provided guidance on the process of selecting and developing a Community Advisory Board and administering meetings. The study team tried to include a broad representation of food and nutrition stakeholders in the community with diverse expertise, perspectives, and experiences.

The Delphi method was implemented with the Community Advisory Board to determine priorities of a dietary intervention research on prevention of obesity and diet-related disease as well as its cultural appropriateness and feasibility. The goal of using the Delphi method was to gather the opinions and priorities of local food and nutrition stakeholders in order to co-design and implement an intervention that is place-based and culturally appropriate for the specific tribal context of the Flathead Reservation. The Delphi method proceeded with a series of focus group interviews to identify the priority populations, the priority health concerns, culturally-relevant intervention methods, the intervention setting and duration, measurement of outcomes, and data analysis.

The first Community Advisory Board focus group served to identify the priority populations and the priority health concerns through a series of prompts provided by the study team to the Community Advisory Board. After identifying the priority populations and the priority health concerns of each stakeholder, the facilitators then worked to prioritize these through rating. The second meeting focused on taking the priority population and health areas that received the highest priority ratings to co-designing solutions and then to translate these solutions into an intervention in-order to collect evidence to back up the co-designed solutions. Specifically, low-income households registered for the Food Distribution Program on Indian Reservations were identified as the most vulnerable priority population with regards to diet-related chronic disease. Diet-related chronic disease and a need to improve dietary quality were identified as the priority health areas. *Eat Fresh* was co-designed to eliminate access barriers to affordable fresh and desirable produce through food and nutrition education, culinary training, and the provision of fresh fruits and vegetables. The third meeting focused on designing the specifics of the intervention including selecting appropriate outcome measures that the Community Advisory Board was comfortable with. A fourth meeting occurred after the intervention to discuss data analysis, implications of findings in the context of the community, and suggestions for a future intervention.

The resulting intervention was a 6-week community-based intervention that provided participants with produce along with weekly in-person food, nutrition, and cooking education in order to improve dietary quality. The intervention was tailored to meet the specific needs of community residents through culturally appropriate programming (30, 31) that included the recognition of local food preferences, as well as constraints of time, financial resources, and access to cooking equipment.

### Sampling

The *Eat Fresh* dietary intervention involved a quasi-experimental evaluation design. Study participants of *Eat Fresh* included low-income households registered for the Food Distribution Program on Indian Reservations (FDPIR; also known as the “Commodities Program”) on the Flathead Reservation. The FDPIR is a federal food assistance program administered by the U.S. Department of Agriculture (USDA) and locally administered by state and tribal organizations (12). Eligible low-income households living on reservations and families with tribal affiliation residing in designated areas near reservations can receive food assistance from the FDPIR as an alternative to the Supplemental Nutrition Assistance Program, another federal food assistance program in the United States (12).

The participants comprised of a convenience sample that were recruited using word of mouth, posters, and mailing of postcards to FDPIR participants. A total of 23 low-income participants signed up for the intervention and 19 participants completed the intervention. Inclusion criteria to participate in the study included: (1) enrollment in FDPIR, (2) resident of the Flathead Reservation, (3) 18 years of age or above and, (4) only one resident per household can participate in the study. Participants were classified as young (19–39 years of age), middle age (40–54 years of age), and older (55+).

Approval for the participation for the involvement of human subjects was received by the Institutional Review Board (IRB) of Salish and Kootenai College and Montana State University. Participants received a $50 incentive weekly for participating in the intervention which included time to prepare recipes at home, for a total of $300 during the course of the intervention.

### Intervention Implementation

Participants received weekly boxes of fresh fruits and vegetables over the 6-week study period that were roughly equal in quantity to the recommended serving sizes of fruits and vegetables by the Dietary Guidelines of Americans per week based on the household size. The study team tried to procure fruits and vegetables that were in season in addition to commonly consumed and locally-desired non-seasonal fruits and vegetables that are available all year including: apples, bananas, blueberries, cantaloupe, cherries, grapefruit, grapes, oranges, peaches, pears, avocados, beets, bell peppers, broccoli, carrots, cauliflower, cucumbers, eggplant, garlic, green beans, kale, lettuce, onions, peas, pumpkin, romaine lettuce, spinach, turnips, zucchini, squash, sweet potatoes, tomatoes, and yams.

The in-person education component took place at Salish Kootenai College, the local tribal college that administered the study, and was implemented by study administrators and staff trained in food, nutrition, and cooking. *Eat Fresh* targeted individual and household food and nutrition knowledge, attitudes, behaviors, and skills focused on preparing meals with fresh fruits and vegetables. Food and nutrition education topics included: (1) dietary benefits of FV consumption, (2) phytochemicals in fruits and vegetable, (3) dietary fiber, (4) plant and animal protein, (5) omega-3 fatty acids, (6) sugar consumption and the glycemic index, (7) eating and blood pressure, inflammation, diabetes, and heart disease, (8) portion control, (9) meal planning and budgeting at the grocery store, (10) making healthy soups, salads, and snacks, and (11) preserving fruits and vegetables.

### Intervention Measures

At pre-intervention, a survey was implemented to collect basic demographic information of participants including their age, education, income level, race, and township. In addition, household food security status was measured by the U.S. Adult Food Security Survey Module: Six-Item Short Form (32). The impacts of *Eat Fresh* were evaluated by collecting the following participant data at pre-intervention (week 0) and at post-intervention (week 6): (1) food procurement practices and perceptions, (2) dietary quality as measured by the Healthy Eating Index (HEI) Scores based on the 2010 Dietary Guidelines, (3) anthropometric measures of body weight, Body Mass Index (BMI), and blood pressure, and (4) perceptions of health.

### Household Food Security Status

Food security status was measured by the U.S. Adult Food Security Survey Module: Six-Item Short Form (32). Based on responses to the Six-Item Short Form, food security status is assigned on a scale from 0 to 6 as follows: (1) score of 0–1: high or marginal food security (2) score of 2–4: low food security and, (3) score of 5–6: very low food security.

### Survey on Food Practices and Perceptions

A structured survey was administered at pre-intervention that asked participants about food procurement and preparation practices. A series of structured surveys were administered weekly throughout the intervention to characterize variation in the following: (1) FV consumption behaviors, (2) other food consumption behaviors and, (3) perceptions regarding the intervention. The survey tools were designed based on input from members of the project's Community Advisory Board and was pilot tested with sample group of community members and refined before being implemented for the study.

### 24-h Dietary Recall Data and Healthy Eating Index (HEI) Scores

Self-reported 24-h dietary recall data were collected using The Dietary Assessment Primer of the National Cancer Institute (33) by a trained researcher. Data from the 24-h dietary recall were entered into Nutritionist Pro Diet Analysis (Axxya Systems) to evaluate energy and nutrient composition of diets at pre-intervention and at post-intervention. This data was then used to calculate Healthy Eating Index-2010 (34, 35) scores to assess how participants' diets were aligned with the 2010 Dietary Guidelines for Americans and if and how this varies between the pre-intervention and post-intervention. Each food item reported in the individual 24-dietary recalls was assigned a USDA food code or ingredient code based on the item description and food group composition using Food Patterns Equivalents Database 2011–12 (36). This procedure resulted in an overall diet quality index made up of the aforementioned 12 components of the HEI for a total of 100 points (34). Each of the following food components can receive a maximum score of 5 in the HEI: (1) total vegetables, (2) greens and beans, (3) total fruit, (4) whole fruit, (5) seafood and plant proteins, and (6) total protein foods. Each of the following food components can receive a maximum score of 10 in the HEI: (1) whole grains, (2) low-fat dairy, (3) fatty acid ratio, (4) refined grains, and (5) sodium. Empty calories can earn a maximum of 20 points. With the exception of the fatty acid ratio, HEI scores use standards that are expressed as a percent of calories or per 1,000 calories. In this study, we calculated HEI component and total scores using published SAS code (37) (version 9.4 SAS Institute Inc., Cary, NC).

### Body Mass Index (BMI) and Blood Pressure

Body Mass Index (BMI) values of study participants were calculated on the basis of weight and height using anthropometric scales [Seca] and a stadiometer [Seca]. The BMI (kg/m2) classification of the National Heart, Lung, and Blood Institute of the National Institutes of Health was applied as follows: (1) underweight <18.5, (2) normal = 18.5–24.9, (3) overweight = 25.0–29.9, (4) obesity class I = 30.0–34.9, (5) obesity class II = 35.0–39.9, and (6) extreme obesity (obesity class III) = 40.0 and above (38).

Systolic blood pressure (SBP) and diastolic blood pressure (DBP) of participants was taken using a sphygmomanometer (Phillips SureSigns VSi; Cambridge, Massachusetts, USA) by a trained technician of tribal affiliation under standard procedure on the on the upper arm while the participant was sitting upright with legs uncrossed. The results were grouped into the classification scheme for adults of the National Heart, Lung, and Blood Institute (38) using the following levels: (1) normal is SBP <120 or DBP <80, (2) prehypertension is SBP 120–139 or 80–89, (3) stage 1 hypertension is SBP 140–159 or DBP 90–99, and (4) stage 2 hypertension is SBP ≥ 160 or DBP ≥ 100.

### Survey on Perceptions of Health

A structured survey on health perceptions was administered at pre-intervention, weekly throughout the intervention, and at post-intervention that asked participants regarding the following perceptions: (1) overall well-being, (2) mood, (3) optimism, (4) mental alertness, (5) energy, (6) weight, (7) flatulence, (8) bowel movements, (9) the way clothes fit, and (10) skin. Participants were also asked if they perceived that FV consumption impacted their health, mood, energy levels, and mental alertness on a scale from 1–5 with 5 being the most impactful.

### Statistical Analysis

Data was analyzed through various statistical tests using JMP (version 12.0 SAS Institute Inc., Cary, NC) and SAS (version 9.4 SAS Institute Inc., Cary, NC). Descriptive statistics were calculated to quantitatively describe population demographics, perceptions of health, and food procurement patterns. A one-way analysis of variance (ANOVA) was applied to examine differences between food security status, HEI Total Scores, body weight, BMI, and blood pressure between pre-intervention and post-intervention. In addition, ANOVA was applied to examine variation of measures based on age. We did not compare between gender and ethnicity because of the uneven number of participants.

## Results

### Participant Demographics

The majority of participants identified as having tribal affiliation (75%) including Salish, Kootenai, Cree, Cherokee/Pache, Blackfeet, Spokane, Shoshore-Bannock, Pembra Band/Chippewa (Please see Supplementary Table 1 for a summary of findings). The majority of participants identified as either Salish or Kootenai (8 participants). The remaining participants identified as being Caucasian (25%). The majority of participants grew up on the Flathead Reservation and lived there for an average of 23 years. Of the 19 participants who completed the study, 16 were female and 3 were male. The average participant age was 48 years and ranged from 19 to 73 years of age. The household size of participants ranged from one to four members with an average of 2 members in a household. A total of 46.15% reported having diabetes, 30.77% reported having heart problems, and two participants identified as having food allergies.

In addition to participating in the FDPIR, some participants also participate other food assistance program including assistance from food banks (37.5% of participants). Few participants have household members that receive senior meals (6.25%) or support from the Special Supplemental Nutrition Program for Women, Infants, and Children (WIC) (5%), the reduced or free school lunch program (10.5%), and the free breakfast for children program (10.5%). The majority of participants (84%) primarily use their own car for transportation while the remaining share rides with family and friends along with the local bus system.

### Food Security Status

The average food security score at pre-intervention on the 6-point scale was 2.95, representing low food security (score of 0–1: high or marginal food security; score of 2–4: low food security and, score of 5–6: very low food security). On average, food security scores were found to be classified as low food security for all age groups and were highest for younger participants (2.33 ± 1.19) followed by older participants (2.75 ± 0.73) and middle-aged participants (3.00 ± 0.38), although differences in means of food security scores based on age was not significant.

### Survey on Food Practices and Perceptions

All participants reported to have a refrigerator, microwave, stove, and cooking utensils and, 95% of participants have a freezer. At pre-intervention, the main barriers identified regarding preparing meals from scratch were: (1) cooking knowledge (42.11%), (2) time to cook (42.11%), (3) cooking skills (21.05%) and, (4) support from household members to eat meals prepared by the participant (15.79%). Access to a kitchen was not recognized by participants as a barrier regarding preparing meals from scratch. Some participants shared that access to better kitchen equipment including a better stove, microwave, freezer, and lighting would make preparing meals from scratch less challenging.

In addition to procuring food from the FDPIR, the majority of participants purchase food from big box stores, grocery stores, and food banks. One participant purchases food from a food cooperative. A total of 35% of participants buy groceries twice or more a week, 20% buy groceries every other week, 15% buy groceries weekly, and 15% buy groceries monthly. The remaining participants noted that they buy groceries as needed or when they have money. On average, participants travel 12 miles to purchase groceries and spend an average of 68 min on grocery shopping per trip. The majority of participants (55%) spend between $50–$99 on groceries for their household on a weekly basis while 20% of participants spend < $50 weekly and 20% spend $100–$199 weekly. One participant spends above $400 weekly on groceries for their household.

On average, participants reporting spending just over an hour (69 min) on meal preparation before the intervention. The majority of participants (84.21%) frequently prepare meals from scratch using mainly basic whole ingredients such as vegetables (fresh, frozen, or canned), raw meats, and rice, for themselves and their household. When preparing meals, the majority of participants (65%) usually prepare meals from memory while the remaining reported that they usually prepare meals from a cookbook or from an online recipe. The mean number of sit-down meals prepared before the intervention per week was 20 (± 8.15). No significant differences were found for number of sit-down meals prepared and consumed between the pre-intervention period, intervention periods, and post-intervention (*p* > 0.60).

**Figure 1:**
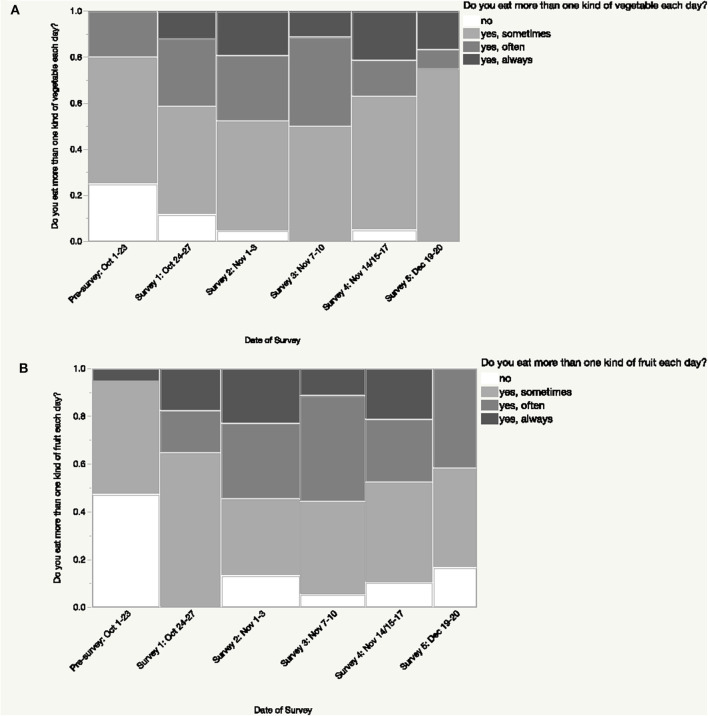
Changes in number of types of fruits and vegetables consumed each day during the intervention. Participants reported consuming a greater number of types of fruits and vegetables daily during the intervention; this difference was significant (*p* < 0.005) for fruits **(A)** but not for vegetables (*p* > 0.19) **(B)**.

**Figure 2:**
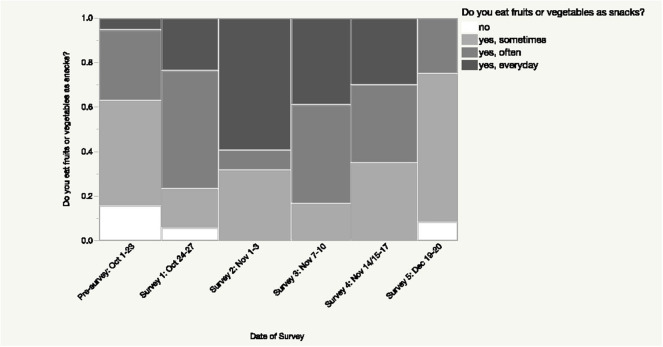
Changes in consumption of fruits and vegetables as snacks during the intervention. A significant difference (*p* < 0.0009) was found in participants' responses regarding their consumption of fruits and vegetables as snacks during the intervention.

Overall, participant consumption of more than one kind of fruit each day increased over the course of the intervention and this change was significant (*p* < 0.005; Figure 1). Notably, at pre-intervention, 47.37% of participants reported that they do not consume more than one kind of fruit each day while at post-intervention this decreased to 16.67% of participants (Figure 1). While participant intake of number of types of vegetables consumed daily increased overall during the intervention, this change was not significant (*p* > 0.19; Figure 1). During the intervention, participants generally reported increasing their consumption of fruits and vegetables as snacks, with significant differences across the intervention (*p* < 0.0009; Figure 2).

In addition to changes in FV consumption, participants reported changes in their beverage consumption. The percentage of participants who reported consuming soda regularly decreased from 55% of participants at pre-intervention to 33.33% at post-intervention while consumption of sugar-sweetened fruit drinks decreased from 60% at pre-intervention to 16.67% at post-intervention. Concurrently, the percentage of participants who use food nutrition labels when shopping increased from 72.41% at pre-intervention to 86.21% at post-intervention.

On a scale from 1–5 regarding how challenging the participants found the intervention to adhere to, with 5 being the most challenging, the average score across the intervention periods regarding was 2.73. Averaging weekly intervention survey responses, a total of 13.95% of responses indicated the intervention was not challenging to adhere to (score of 1), 27.91% of responses were a score of 2, 33.72% of responses with a score of 3, 19.77% responses were a score of 4, and 4.65% of responses found the intervention very challenging to adhere to (score of 5). On a scale from 1–5 regarding how well the intervention recipes met taste preferences, with 5 most meeting taste preferences, the average score across the intervention was 4.23. When combining the weekly intervention survey responses, 47.67% of responses indicated the recipes strongly met taste preferences (score of 5), 32.56% of responses were a score of 4, 16.26% of responses were a score of 3, 2.33% of responses were a 2, and only 1% of the responses had a score of 1 that the recipes did not meet taste preferences.

Combining the weekly survey responses regarding barriers to using the fruits and vegetables provided by the *Eat Fresh* intervention, the main barriers were as follows: (1) access to other ingredients (39.51% of responses), (2) time to cook (35.80%), (3) lack of financial resources to procure ingredients (33.33%), (4) cooking knowledge (28.40%), (5) cooking skills (27.16%), and (6) lack of proper kitchen equipment (20.99%). Some participants noted difficulty of the recipes provided by the intervention (14.81%), lack of support for cooking from their household (13.58% of responses), greater personal motivation to consume fruits and vegetables (13.58% of responses), and that the recipes would be easier to follow if they were more appealing (11.11% of responses). The most prevalent kitchen equipment that participants noted that they would like to have for cooking include a food processor, stove with oven, and chopper. Some participants would also like a juicer, blender, and a slow cooker.

### Healthy Eating Index Scores

**Figure 3:**
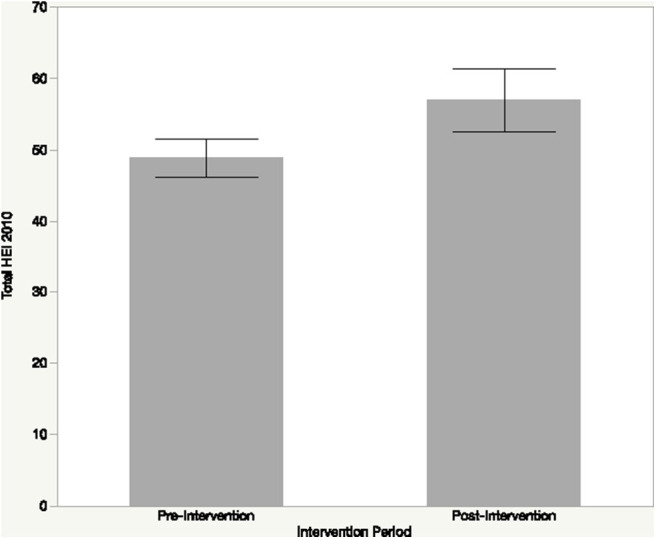
Healthy eating index (HEI-2010) scores of study participants at pre- and post-intervention. Dietary quality based on the HEI improved during the intervention, however, this change was not statistically significant (*p* > 0.12).

The mean HEI score of participants was 48.82 (± 11.88) out of 100 during the pre-intervention period and 56.92 (± 11.88) out of 100 during the post-intervention period (Figure 3 and Table 1). However, this improvement in overall dietary quality was not statistically significant (*p* > 0.12).

**Table 1:** HEI-2010 component and total scores at pre- and post-intervention (n = 20).

**Component**	**Maximum value**	**Standard for maximum score**	**Standard for minimum score of zero**	**Pre mean (SD)**	**Post mean (SD)**	***p*-value**
**Total fruit^a^**	5	≥0.8 cup equivalent per 1,000 kcal	No fruit	1.7 (1.8)	3.0 (2.2)	0.0201^*^
**Whole fruit^b^**	5	≥0.4 cup equivalent per 1,000 kcal	No whole fruit	2.5 (2.0)	3.0 (2.4)	0.3843
**Total vegetables^c^**	5	≥1.1 cup equivalents per 1,000 kcal	No vegetables	3.0 (2.0)	2.9 (1.9)	0.8392
**Greens and beans^c^**	5	≥0.2 cup equivalent per 1,000 kcal	No dark green vegetables or beans and peas	0.9 (1.9)	2.2 (2.4)	0.0759
**Whole grains**	10	≥1.5 oz equivalents per 1,000 kcal	No whole grains	2.9 (3.5)	3.8 (4.5)	0.2757
**Dairy^d^**	10	≥1.3 cup equivalents per 1,000 kcal	No dairy	5.9 (2.8)	5.9 (3.8)	0.9966
**Total protein foods^e^**	5	≥2.5 oz equivalents per 1,000 kcal	No protein foods	4.2 (1.3)	4.2 (1.5)	0.9441
**Seafood and plant proteins^e^^,^^f^**	5	≥0.8 oz equivalent per 1,000 kcal	No seafood or plant proteins	2.1 (2.3)	1.9 (2.4)	0.6900
**Fatty acids^g^**	10	(PUFAs + MUFAs)/SFAs > 2.5	(PUFAs + MUFAs)/SFAs ≤ 1.2	3.5 (3.5)	5.5 (4.1)	0.0654
**Refined grains**	10	≤ 1.8 oz equivalents per 1,000 kcal	≥4.3 oz equivalents per 1,000 kcal	7.1 (3.0)	6.1 (4.5)	0.4826
**Sodium**	10	≤ 1.1 g per 1,000 kcal	≥2.0 g per 1,000 kcal	2.8 (3.0)	3.5 (3.9)	0.5162
**Empty calories^h^**	20	≤ 19% of energy	≥50% of energy	12 (6.6)	15 (6.4)	0.2598
**Total**	100			49 (12)	57 (20)	0.1355

* *p <0.05*.

a *Includes fruit juice*.

b *Includes all forms except juice*.

c *Includes any beans and peas not counted as Total Protein Foods*.

d *Includes all milk products, such as fluid milk, yogurt, and cheese, and fortified soy beverages*.

e *Beans and peas are included here (and not with vegetables) when the Total Protein Foods standard is otherwise not met*.

f *Includes seafood, nuts, seeds, soy products (other than beverages) as well as beans, and peas counted as Total Protein Foods*.

g *Ratio of polyunsaturated fatty acids (PUFAs) and monounsaturated fatty acids (MUFAs) to saturated fatty acids (SFAs)*.

h *Calories from solid fats, alcohol, and added sugars; threshold for counting alcohol is > 13 g/1,000 kcal*.

Specifically, for HEI component scores, total fruit increased from 1.69 (out of 5 points) during the pre-intervention to 2.96 during the post-intervention with significant differences (*p* < 0.05) between intervention periods. HEI scores for whole fruits increased from 2.46 (out of 5) points during the pre-intervention to 3.02 during the post-intervention; however, this difference was not significant (*p* > 0.43). HEI scores for total vegetables showed little change from 3.03 (out of 5) points during the pre-intervention to 2.89 points during the post-intervention without a significant difference between intervention periods (*p* > 0.83). HEI scores for greens and beans notably increased from 0.91 (out of 5 points) during the pre-intervention to 2.18 during the post-intervention, the difference was not significant (*p* > 0.07). Several additional food groups that are required for healthy eating patterns based on the Dietary Guidelines for Americans showed improvement across the intervention periods including whole grains and fatty acids while indicating a decrease in sodium and empty calories (Table 1), although these improvements were not statistically significant.

### Body Mass Index (BMI) and Blood Pressure

Overall, participants had a mean BMI of 34.42 (± 10.07) at pre-intervention which is classified as obesity class I. This included four participants with a BMI classified as normal, three overweight, three obesity class I, four obesity class II, and five extreme obesity. At post-intervention, participants had a mean BMI of 34.58 (± 9.88) during the post-intervention period which is classified as obesity class I, the same level as that during pre-intervention, with no significant differences (*p* > 0.90). This included three participants had a BMI classified as normal, four overweight, three obesity class I, four obesity class II, and five extreme obesity. Thus, one participant increased BMI from normal to overweight during the intervention. However, while the BMI classification of one participant increased when comparing between pre- and post-intervention, the BMI values of seven participants increased while the BMI decreased for 8 participants and stayed the same for the remaining.

Participants had a mean systolic and diastolic blood pressure of 127.10 mm Hg (± 10.99) and 81.84 mm Hg (± 4.31), respectively, at pre-intervention, which is classified as prehypertension. Similarly, participants had a mean systolic and diastolic blood pressure of 128.44 mm Hg (± 12.42) and 83.50 mm Hg (± 4.97), respectively, at post-intervention, which is classified as prehypertension. Overall, eight participants experienced a decrease in blood pressure during the intervention, ten participants experienced an increase in blood pressure during the intervention, and one participant had no change in blood pressure. However, changes in blood pressure was not significant during the intervention (*p* > 0.96).

### Perceptions of Health

Multiple participants noted that they experienced improvements during the *Eat Fresh* intervention in various self-reported health parameters including their overall perceived well-being, mood, optimism, mental alertness, energy, feelings regarding weight, and skin (Table 2). When averaging across the weekly data during the intervention, a notable percentage of participants who completed the survey perceived an increase in energy during the intervention (59% of participants) as well as improvements in overall well-being (46%) and mood (44%). At post-intervention, the majority (83%) of participants who completed the survey perceived an improvement in energy levels based on the intervention. In addition, half of the participants perceived an improvement in mood at post-intervention while 42% of participants perceived an improvement in overall well-being.

**Table 2:** Perceived changes in self-reported health parameters across the *Eat Fresh* intervention.

**Perceived changes with *Eat Fresh* intervention**	**Intervention**	**Intervention**	**Intervention**	**Intervention**	**Average during**	**Post-intervention**
	**week 2**	**week 3**	**week 4**	**week 5**	**intervention**	
#respondents who completed the survey	17	22	18	20	19.25	12
Improvement in overall well-being	41%	36%	56%	50%	46%	42%
Improvement in mood	35%	27%	50%	65%	44%	50%
Increased optimism	35%	5%	39%	20%	25%	0%
Increased mental alertness	24%	36%	33%	25%	30%	8%
Increased energy	53%	64%	50%	70%	59%	83%
Improved feelings regarding weight	18%	14%	11%	35%	19%	8%
Changes in flatulence	12%	14%	11%	5%	10%	8%
Changes in bowel movements	29%	41%	39%	50%	40%	42%
Changes in the way clothes fit	6%	14%	28%	20%	17%	25%
Improvement in skin	24%	14%	22%	0%	15%	17%

When asked about how eating the fruits and vegetables provided by the *Eat Fresh* intervention make them feel about their health, a majority of participants responded that FV consumption made them feel either very good (51.16%) or good about their health (43.02%) when combining the weekly intervention survey data. The two most prevalent themes from the open-ended responses regarding how eating fruits and vegetables impacted health was improved energy levels and improved mental well-being. For example, one participant stated, “I have more energy and awareness” during the post-intervention survey and another stated, “Eating right gave me a positive mental attitude.”

When asked about how eating the fruits and vegetables provided by the *Eat Fresh* intervention made them feel about their mood on a scale of 1–5 with 5 being the happiest, the majority of responses were either a score of 5 (42.68%) or 4 (41.46%) with some scores of 3 (14.63%), only 1.22% responses of a score of 2, and no scores of 1. Similarly, when asked about how eating the fruits and vegetables provided by the *Eat Fresh* intervention made them feel about their energy levels on a scale of 1 to 5 with 5 being the most energetic, the majority of responses were either a score of 5 (36.36%) or 4 (46.59%) with some scores of 3 (17.05%), and no scores of 2 or 1.

## Discussion

Findings from the *Eat Fresh* pilot study highlight both intended and unintended consequences of a well-intentioned community-based fruit and vegetable (FV) intervention targeted at increasing produce consumption toward improving dietary quality and perceptions of health. Intended consequences of *Eat Fresh* aligned to project goals including improvements in the number of types of FVs consumed daily, servings of FVs consumed, improvements in dietary quality, and improvements in participant perceptions of well-being including overall well-being, mood, optimism, mental alertness, energy, feelings regarding weight, and skin. While the *Eat Fresh* pilot study was successful in meeting its goal of enhancing FV consumption and associated perceptions of well-being, it also resulted in several unintended negative consequences as well as presented challenges which provide important lessons for designing future interventions.

Specifically, seven participants (out of 19 participants) had an increase in BMI values between the pre- and post-intervention periods; however, this BMI change was not statistically significant and the BMI classification stayed the same for 6 of these participants. Additionally, ten participants had an increase in blood pressure at post-intervention; however, this increase in BMI was not significant and the blood pressure classification stayed the same for 6 out of these participants. Findings further highlight barriers and challenges to adhering to dietary interventions associated with preparing meals from whole foods. Study participants found *Eat Fresh* moderately challenging to adhere to with barriers of access to ingredients, time constraints to cook, lack of financial resources to procure ingredients, cooking knowledge, and cooking skills. The shortcomings and unintended consequences of *Eat Fresh* are attributed to the lack of a systems approach (39) where a focus on one part of the diet may pose risks to other parts of the diet and food system more broadly. The shortcomings of *Eat Fresh* provide insight on designing future dietary interventions that take a systems approach aimed at improving dietary quality (16, 40, 41).

This pilot study provides evidence that study participants on the Flathead Reservation have diet-related disparities with relatively low food security scores compared to the national average as well as dietary quality with regards to national dietary recommendations. The prevalence of low food security scores among participants, who primarily comprise of tribally-affiliated members residing on the Flathead Reservation, is in line with previous research regarding diet-related health disparities among Native American communities (15, 16, 18, 20–22). The food security status of study participants shows a marked health disparity compared to the national average in the United States (42). Low food security is directly linked to poor dietary quality including decrease in consumption of the quantity and diversity of healthy foods including fruits and vegetables coupled with an increase in the consumption of energy-dense foods such as ultra-processed foods and those high in sugar and fat (43–46). Findings from this pilot study further highlight the overall low dietary quality of participants with a mean HEI of 48.82 (± 11.88) out of 100. These dietary quality findings are congruent with that of low-income adults in the United States that have been shown to have mean HEI scores of 45.4 (47) compared to the total population of the United States that has a HEI total score of 59 (35). These findings are further in line with previous research on the Flathead Reservation focused on dietary quality of residents (15). The low HEI scores for FVs and other foods that are recommended for a healthy diet at the pre-intervention period of *Eat Fresh* was on par with populations in the United States and globally who overall are consuming more calories than needed, but not adequate amount of calories from recommended food groups including nutrient-dense foods (48).

Results of *Eat Fresh* highlight how a FV intervention can improve dietary quality over the short-term toward meeting national dietary recommendations with HEI scores increasing during the intervention from 48.82 to 56.92, however this improvement not statistically significant. The improvements in HEI during the intervention are linked to increased consumption of FVs that were promoted by *Eat Fresh* along with other healthy foods including whole grains and fatty acids. While the increase in HEI scores for total fruit consumption was statistically significantly between the pre- and post-intervention periods, the increase in HEI scores for greens and beans was not statistically significant. One reason for the difference in significance between fruits and vegetables is likely linked to the barriers participants noted in preparing foods with FVs; fruits can generally be eaten without preparation whereas vegetables generally require preparation for desirable consumption. Future research is called for to examine if improvements in dietary quality are sustainable for the long-term beyond the intervention duration, particularly in the context of the challenges that participants noted with regards to adhering to the intervention.

The improvements in dietary quality with the implementation of *Eat Fresh* were aligned to positive influences on multiple self-reported and perceived parameters of well-being including improvements in perceived overall well-being, mood, optimism, mental alertness, energy, feelings regarding weight, and skin. Almost all participants agreed that the FVs provided by the *Eat Fresh* intervention made them feel good or very good about their health as well as made them feel happy or very happy. These findings are in line with previous studies that provide evidence on the linkage of FV consumption with perceptions of happiness, life satisfaction, and well-being within a 2-year period (49). While the physiological health benefits of healthy eating accrue and are felt decades later, the perceived well-being improvements from increased consumption of FVs are closer to immediate (49). In addition to perceived well-being improvements, previous studies have shown significant short-term improvements to participant's psychological well-being within a 14-day period on the basis of provisioning of FVs to 171 young adults (50). The self-reported health measures point to the importance for food and nutrition interventions to evaluate participant perceptions of health in addition to anthropometric measures in order to capture the multi-dimensionality of well-being.

While *Eat Fresh* met its goal for enhancing FV consumption, multiple barriers were noted by participants in preparing and consuming meals with FVs and adhering to healthy diets which can provide important lessons for designing future dietary interventions in low-income, rural, and tribal communities. Overall, participants found *Eat Fresh* moderately challenging to adhere to with the main barriers being access to ingredients in recipes, time constraints to cook, lack of financial resources to procure ingredients, cooking knowledge, and cooking skills. These challenges are expected to be exacerbated outside of the duration of the intervention when participants are not provided weekly provisioning of fresh fruits and vegetables. Future intervention design should take into account these barriers including providing simpler recipes with fewer and more accessible ingredients that require less time and cooking skills to prepare. However, these recipes should be tested in the community to meet local taste preferences while having accessible ingredients; the majority of participants reported intervention recipes met taste preferences which likely supported the enhancements in FV consumption. The community-engaged approach of designing the intervention through focus group meetings with a Community Advisory Board of food and nutrition stakeholders played a key role in supporting the desirability of the provided foods and recipes among participants. In meeting local taste preferences, future FV interventions should take a place-based approach such as incorporating indigenous foods in tribal communities including wild game, fish, and edible plants into interventions should be considered (51). Training on prioritizing time and scheduling on procuring and preparing meals can further increase the capacity to prepare healthy meals from scratch (52). For example, meal planning has been shown to be a promising tool to offset time scarcity and lead to healthier diets and less obesity (52). Low income is another critical barrier to supporting healthy diets; lower income is associated with cheaper and more energy-dense food choices (53). Diet optimization techniques that are sensitive to cost and social norms can help overcome this barrier by identifying affordable, desirable, and nutrient-rich foods (53). At the same time, there is a need for systemic change that support livelihoods in addition to programs and policies focused on healthy and sustainable food environments and diets.

While *Eat Fresh* led to intended consequences during the intervention period, it did result in several unintended consequences including the increase in BMI and blood pressure for several participants. The increase in BMI and blood pressure can be attributed to focus on *Eat Fresh* in promoting the increased consumption of FVs within healthy diets rather than a whole-diets approach where portions sizes and the consumption of unhealthy foods were restricted. While the consumption of unhealthy foods and portion size were touched upon during the in-person classes, these topics were not the focus of the *Eat Fresh* nor was the consumption of unhealthy foods restricted during the intervention. Multiple participants shared that the recipes with the provided FVs did not satiate their appetite and thus they ate additional snack foods or meals. Future interventions that promote the consumption of FVs should thus consider dietary restrictions or recommendations on consumption of unhealthy foods in ways that are culturally appropriate. Previous studies have highlighted that interventions to promote healthy eating as a way to achieve and maintain healthy weights do not work for most people and in cases, have been linked to negative unintended consequences including social, psychological and economic costs (54, 55) such as loss of self-efficacy, self-esteem issues, the ability to cope with barriers to healthy eating, body dissatisfaction and social stigma, self-blame, discouragement, discomfort eating with others including attending community events, and issues with household budgets (55). In addition, previous work has highlighted the potential harm of simple dietary recommendations in having negative unintended consequences and the need and power of creative ways to extend nutrition advice that is actionable, balanced and avoids unintended and unhealthy consequences while sustaining health over the long term (56).

Based on findings from this pilot study, the research team and members of the Community Advisory Board have co-designed a more holistic dietary intervention, Healthy and Sustainable Diets for All, to follow *Eat Fresh* that takes a place-based approach incorporating themes of sustainable diets and indigenous food systems to support both human and planetary health (16). As part of this on-going intervention, the study team is incorporating dietary restrictions and education about unhealthy foods including limiting the consumption of ultra-processed foods.

This pilot study had multiple limitations which can be addressed in designing future community-based interventions. Two major limitations are the small sample size of 19 participants as well as the short duration of the intervention of 6 weeks. The small sample size of the study does not allow us to expand the findings beyond the participants of the study and may further have had an impact on the relative magnitude of unintended consequences in the study. Additional limitations of this pilot study include the use of self-reported dietary recall data for two single days at pre- and post-intervention which may not reflect actual behaviors or the variation that occurs in diets day-to-day as well as at different seasons or times of the month. Future research is called for that builds on the results illuminated through the *Eat Fresh* pilot study using a larger sample size and a longer duration intervention. Data from such future work should be analyzed on the basis on multiple socio-economic demographics to better understand the social determinants of health. Future research should also measure additional biomarkers to evaluate the impacts of the intervention to supplement the self-reported dietary recall data. In addition, future research should make not of any unintended consequences such as those reported here that are largely missing from the literature yet have importance for practical and ethical reasons. Lastly, as a large percentage of the study participants purchased food in addition to the food provided by *Eat Fresh* and the FDPIR (Commodities Program), future research should explore how the amount of funds spent on food impact participant study outcomes. Overall, findings from this pilot study add to the emerging literature on the relationship between FV consumption, dietary quality and well-being in health disparate communities, as well as the small body of literature that reports on the negative unintended consequences of a food and nutrition intervention.

## Conclusion

Findings highlight both intended and unintended consequences of a dietary intervention that can be applied to design future community-based programs for improving dietary quality and plant-based diets while addressing health disparities in tribal and rural communities. The unintended consequence of *Eat Fresh* is primarily attributed to the lack of a systems approach (39). As plant-based diets are increasingly recommended for supporting human and planetary health (28), several key lessons can be adapted from *Eat Fresh* to inform the co-design of evidence-based interventions in communities for supporting public health: (1) it is important for dietary interventions to be holistic and focus on the whole diet instead of only focusing on the consumption of healthy foods such as FVs; (2) interventions should measure multiple parameters to assess their effectiveness including both objective dietary and health outcomes as well as subjective measures such as participant perceptions of well-being; (3) it is important to collaborate with a Community Advisory Board of food and nutrition stakeholders in co-designing dietary interventions to help ensure the intervention questions, design, outcomes, analysis, and dissemination are place-based and culturally relevant. For example, working with a Community Advisory Board and the Delphi Method can help ensure that the foods and recipes provided to participants of a dietary intervention are affordable, accessible, convenient, and desirable including being culturally appropriate; (4) dietary interventions should be co-designed to be multi-phased and target individual, familial, community, and environmental dimensions including existing community infrastructure in order to have long-term effects and sustainable effects for supporting well-being through diets and; (5) in taking a systems approach, dietary interventions should foster greater linkages between local agricultural sources and diets to enhance local food systems.

## Data Availability Statement

The datasets generated for this study are available on request to the corresponding author.

## Ethics Statement

The studies involving human participants were reviewed and approved by Institutional Review Board at Salish Kootenai College and Institutional Review Board at Montana State University. The patients/participants provided their written informed consent to participate in this study.

## Author Contributions

SA, CB, and MT were responsible for research design. MT and MR collected data. SA, CB, and TG analyzed data and created graphs and tables. All authors interpreted data. SA and CB wrote the manuscript with contributions from all authors. All authors contributed to the article and approved the submitted version.

## Conflict of Interest

The authors declare that the research was conducted in the absence of any commercial or financial relationships that could be construed as a potential conflict of interest.
