# Supplementary material for: Intended and Unintended Consequences of a Community-Based Fresh Fruit and Vegetable Dietary Intervention on the Flathead Reservation of the Confederated Salish and Kootenai Tribes
Source: Front Public Health. 2020 Aug 7;8:331. doi: 10.3389/fpubh.2020.00331 (PMC7426441; doi:10.3389/fpubh.2020.00331)
Supplement: Supplementary file 1 [file Data_Sheet_1.docx]

Supplementary Material

# Supplementary Figure

**Supplementary Figure 1. PRISMA flow diagram.** PRISMA flow diagram depicting the number of articles identified, screened, assessed for eligibility, and included in the systematic review synthesis.

# Supplementary Tables

**Supplementary Table 1.** **Systematic review search terms.** This table lists the terms used in the systematic review literature search to answer the question: *What are effects of environmental variation related to climate change on tea quality?*

**Supplementary Table 2. Effects of seasonality on tea quality**

**Supplementary Table 3. Effects of water stress on tea quality**

**Supplementary Table 4. Effects of geography on tea quality**

**Supplementary Table 5. Effects of light factors on tea quality**

**Supplementary Table 6. Effects of altitude on tea quality**

**Supplementary Table 7. Effects of herbivory and microbes on tea quality**

**Supplementary Table 8. Effects of Temperature on Tea Quality**

**Supplementary Table 9. Soil and nutrient effects on tea quality**
